# Does obesity affect patient-reported outcomes following total knee arthroplasty?

**DOI:** 10.1186/s12891-022-04997-4

**Published:** 2022-01-17

**Authors:** Fatemeh Baghbani-Naghadehi, Susan Armijo-Olivo, Carla M. Prado, Leah Gramlich, Linda J. Woodhouse

**Affiliations:** 1 Faculty of Rehabilitation Medicine, University of Alberta, Edmonton, Alberta Canada; 2 Faculty of Business and Social Sciences, University of Applied Sciences, Osnabrück, Germany; 3 Faculty of Medicine & Dentistry, University of Alberta, Edmonton, AB Canada; 4 Faculty of Agricultural, Food, and Nutritional Science, University of Alberta, Edmonton, Alberta Canada; 5 Tufts University, School of Medicine, Department of Public Health & Community Medicine, Division of Physical Therapy, Boston, Massachusetts USA

**Keywords:** Obesity, Total knee arthroplasty, Osteoarthritis, Pain, Function, Quality of life, Patient-reported outcome measures

## Abstract

**Background:**

There is an existing perception that obesity has a negative impact on complications following total knee arthroplasty (TKA). However, data on the impact of obesity levels on patient-reported outcomes (PROMs) is sparse. We investigated the association between different obesity classes with PROMs among patients who underwent TKA.

**Methods:**

We performed retrospective secondary analyses on data extracted from the total joint replacement data repository (Alberta, Canada) managed by the Alberta Bone and Joint Health Institute (ABJHI). Patients had WOMAC and EQ5D scores measured at baseline in addition to 3 and/or 12 months following TKA. Patients were stratified according to the World Health Organization (WHO) classification, into five body mass index (BMI) groups of normal, overweight, BMI class I, BMI class II, and BMI class III. The association between BMI and mean changes in WOMAC subscales (pain, function, and stiffness) and EQ-5D-5L index over the time intervals of baseline to 3 months and 3 to 12 months following TKA was assessed. Linear mixed-effects models were used, and the models were adjusted for age, sex, length of surgery, comorbidities, year of surgery, and geographical zone where the surgery was performed.

**Results:**

Mean age was 65.5 years (SD = 8.7). Postoperatively, there was a significant improvement (*p* < 0.001) in WOMAC subscales of patient-reported pain, function, and stiffness, as well as EQ-5D-5L regardless of BMI group. Although, patients in BMI class II and class III reported significantly improved pain 3 months after TKA compared to those with normal BMI, all BMI groups attained similar level of pain reduction at 12 months after TKA. The greatest improvement in all WOMAC subscales, as well as EQ5D index, occurred between baseline and 3 months (adjusted *p* < 0.0001).

**Conclusion:**

The findings indicate that patients reported improved pain, function, and stiffness across all BMI groups following TKA. Patients with BMI classified as obese reported similar benefits to those with BMI classified as normal weight. These results may help health care providers to discuss expectations regarding the TKA recovery in terms of pain, function, and quality of life improvements with their TKA candidates.

**Supplementary Information:**

The online version contains supplementary material available at 10.1186/s12891-022-04997-4.

## Background

Osteoarthritis (OA) is a degenerative chronic disease that affects 10–15% of adults in Canada and results in pain, disability, and reduced quality of life [1]. The knee is the most commonly affected joint [2]. When conservative treatments fail, patients are typically offered total knee arthroplasty (TKA), which is a well-established and effective intervention for end-stage OA [3]. The overall treatment goal of TKA is to relieve pain, restore loss of function, and improve the health-related quality of life (HRQoL) [4, 5]. Despite the known benefits of TKA on health-related outcomes, some patients experience complications [2, 6] and may receive less benefit than expected. Patients in the higher spectrum of body mass index (BMI) may be at greater risk of poor outcomes after TKA and surgeons are left unsure as to whether TKA is beneficial for patients with higher BMI [7–9], especially class III.

While some studies suggest that BMI has no impact on postoperative recovery and subsequent pain and function [10, 11], others suggest it has a negative impact [12–18]. The association, if any, between BMI and PROMs following surgery remains unclear [9, 11, 19]. A recently published meta-analyses [20] reported that the discrepancy in the results is related to the fact that most studies did not control for confounding factors such as age and sex, and they used different definitions of obesity. A Workgroup of the American Association of Hip and Knee Surgeons Evidence Based Committee suggested that future studies subclassify BMI using the World Health Organization Classification (WHO) to examine the value of TKA in this population [21].

The purpose of the current study was to evaluate the association between BMI, categorized according to the WHO classification, with Patient-Reported Outcome Measures (PROMs) preoperatively, pre- to 3 months postoperatively, as well as 3 to 12 months after TKA adjusting for putative confounders. We used the Western Ontario and McMaster Universities Osteoarthritis Index (WOMAC) and EuroQol-5D (EQ5D) as PROMs that have been widely used to evaluate the effectiveness of TKA [22–24]. The WOMAC questionnaire was used to measure self-reported pain, stiffness, and function, while the EQ5D questionnaire was used to assess the HRQoL, preoperatively, and again 3 and 12 months following TKA.

## Method

### Data source and sample

This study was a retrospective secondary data analysis using a provincial database in Alberta managed by the Alberta Bone and Joint Health Institute (ABJHI). Patients who underwent primary unilateral TKA between 2012 and 2016 and who completed the Western Ontario and McMaster Universities (WOMAC) Osteoarthritis Index (*N* = 7714), and the patient-reported EuroQol-5D (EQ-5D; *N* = 3848) were included in the study. We also used the discharge abstract database, which is a hospital administrative database that is collected as part of the standardized care process and not part of a clinical study. Diagnosis and procedure coding were based on the 10th version of the International Classification of Diseases combined with the Canadian Classification of Health Intervention (ICD-10-CA/CCI). Standardized care processes and consistent data collection for total joint arthroplasty in the province commenced in 2009 and are ongoing. Knee surgeries are performed at twelve public hospitals (Alberta Health Services: AHS) in Alberta, Canada, and all data on patients who underwent surgery were captured and sent to ABJHI for quality assurance and monitoring under the authority of the provincial Privacy Impact Analysis (PIA) agreement (OIPC File # H2801).

**Fig. 1:**
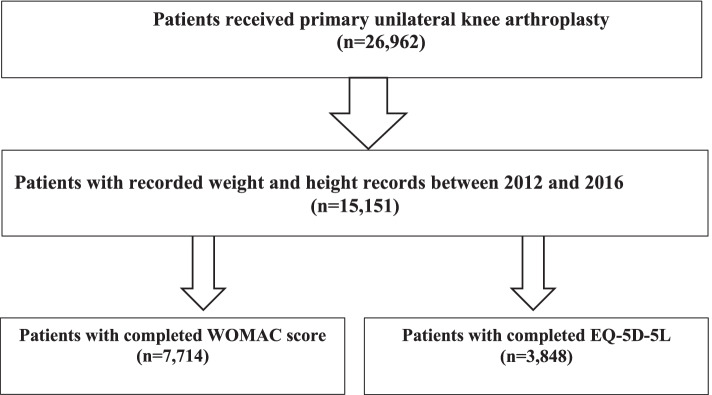
Flowchart for patients’ enrollment and inclusion/exclusion

Between 2012 and 2016, we identified 26,962 patients who underwent primary unilateral TKA from Alberta Bone and Joint Health Repository collected information. A subset of 15,151 patients had height and weight records. Within that group, two separate datasets (7714 patients with WOMAC and 3848 patients with EQ5D questionnaire) were prepared (Fig. 1). BMI was calculated based on weight and height records (measured in each clinic by a nurse) by dividing weight in kilograms (kg) by height in meter squared (m^2^). Patients were then classified into one of five BMI groups according to the World Health Organization (WHO) classification of BMI: normal (BMI ≤ 24.99 kg/m^2^), overweight (25 ≤ BMI ≤ 29.99 kg/m^2^), BMI class I (30 ≤ BMI ≤ 34.99 kg/m^2^), BMI class II (35 ≤ BMI ≤ 39.99 kg/m^2^), and BMI class III (BMI ≥ 40 kg/m^2^) [25]. A total of 9 patients with a BMI lower than 18.5 kg/m^2^ (underweight) were included in the BMI normal group. In addition to WOMAC and EQ5D, information on age, sex, operation time (min), number of comorbidities, perioperative/postoperative complications, year of surgery, and the geographical zone of service were also available. Comorbidities included diabetes, moderate or severe mental health issues, cardiac disease, pulmonary disease, circulatory/clotting disorder, dementia, renal failure, cerebrovascular disease, and moderate or severe liver disease as recorded in the database. Perioperative and postoperative complications were blood transfusion, pulmonary embolism, deep wound infection, myocardial infarction, ileus, pneumonia, deep vein thrombosis, gastrointestinal bleeding, readmission within 30 days, and cerebrovascular accident. Geographical zone of service (where the surgery was performed) included South, Central, and Edmonton. Ethics approval for this study was obtained from the University of Alberta Health Research Ethics Board. Permission to extract the data was obtained from, and done by, ABJHI.

### Patient-reported outcomes

ABJHI uses the WOMAC and EQ5D (described in more detail below) to determine the effectiveness of the TKA surgery. These two outcomes are widely used PROMs in knee arthroplasty settings and are the primary outcomes in the present study.

### WOMAC

The WOMAC Index, developed by Bellamy et al. [23] uses a 5-point Likert scale scale (0 = none, 1 = mild, 2 = moderate, 3 = severe, 4 = extreme) and contains 24 items covering three dimensions of pain (5 items), stiffness (2 items), and function (17 items). A total score combining the three dimensions may be used. Scores range from 0 to 20 for pain, 0-8 for stiffness, 0-68 for physical function, and 0-96 for the total aggregated score. A recent systematic review by Copsey et al. (2019) pointed out that a clear reporting of standardized WOMAC scoring system should be implemented and all subscales should be converted to a 0-100 scale [26]. Using the transformed data has also been recommended in the WOMAC user guide and previous studies [27]. Each of the WOMAC subscales (i.e. pain, stiffness, function) and total score were converted to a scale of 0 to 100 (with 100 being the worst pain, stiffness, or function) by dividing the subscale score by the total possible score and multiplying by 100 [23, 26, 28]. The WOMAC questionnaire is reliable, valid, feasible, and responsive to change over time in people with knee osteoarthritis [23, 29, 30]. The minimal clinically important difference (MCID) values after rehabilitation programs for WOMAC pain, stiffness, and physical function (on the scale of 0-100) were reported as 7.09, 16.2, and 11.25, respectively [31, 32].

### EQ5D

The quality of life of patients before and after surgery was measured using the EQ5D-5 L index, a standardized self-report instrument for measuring generic health status. The EQ5D is widely used in the orthopedic field and medical research to collect HRQoL scores as a basis for determining health status. It has been routinely applied in TKA programs in Alberta, Canada [33]. EQ5D-5 L has good reliability and validity [22, 34, 35], and consists of 5 dimensions including mobility, self-care, usual activities, pain/discomfort, and anxiety/depression. Items are rated from 1 (no problems) to 5 (extreme problems). From these five dimensions, a health utility (EQ5D index) is calculated ranging from − 0.59 to 1.00, with 1.00 indicating full health and 0 representing death. Negative EQ5D scores are possible, and they indicate health status valued worse than death [36]. The MCID for the EQ5D index is reported to be 0.20 [37].

### Statistical analyses

Sample characteristics were presented as mean and standard deviation for continuous variables, and frequencies for categorical variables. The association between each of the main dependent variables with independent variables (BMI, age, sex, number of comorbidities, length of surgery, year of surgery, and geographic zone of service) were determined in univariate fashion using Pearson correlation and t-test when applicable. We examined for multicollinearity among the independent variable(s) using the variance-inflation factor (VIF) to avoid overadjusting. Variance-inflation factor of 4 [38] has been considered as the cut-off criterion to remove predictor variables that are highly correlated, which ensures stability and reliability of the developed model. Repeated measurement analyses using mixed effects models were performed to investigate the association between each of the patient-reported outcome scores (WOMAC and EQ5D) and BMI groups. Separate models were used to analyze each of the dependent variables of pain, stiffness, physical function, total WOMAC scores, and EQ5D index. Since linear mixed effects models consider all available data and thus allow for missing values, any participants who had baseline in addition to 3 and/or 12 months follow-up data were included in the analysis. All models were adjusted for age, sex, number of comorbidities, length of surgery, year of surgery, and geographic zone of service. The patient effect was considered as a random effect in the model. The interaction of time by BMI in the models was considered to adjust for the within subject variation overtime, and the interaction term provides the adjusted mean changes for each group at different time intervals. All statistical analyses were performed using R software package version 0.99.902.

## Results

### Patient characteristics

The mean ± SD BMI of patients who were included in the study was 33.3 ± 6.9 kg/m^2^. Mean age of patients was 65.5 ± 8.7 years old, and 61% of the sample was female. Among all patients, 21.7% had at least one comorbidity, and 13% had at least one complication.

Patient demographic and baseline characteristics for each of the five BMI groups are also given in Table 1. On the basis of BMI, 572 (7.4%) of the participants had BMI normal; 2122 (27.5%) were overweight; 2314 (29.9%), BMI class I; 1460 (19.0%), BMI class II; and 1246 (16.2%), BMI class III.

**Table 1:** Baseline characteristics^a^

	BMI groups according to baseline values (kg/m^**2**^)					
	Overall N = 7714	< 25 Normal ***N*** = 572 (7.4%)	25-29.9 Overweight ***N*** = 2122 (27.5%)	30-34.9 Obese class I ***N*** = 2314 (29.9%)	35-39.9 Obese class II ***N*** = 1460 (19.0%)	***≥***40 Obese class III ***N*** = 1246 (16.2%)
**BMI (kg/m**^**2**^**)**	33.3 ± 6.9	23.0 ± 1.6	27.7 ± 1.4	32.3 ± 1.4	37.2 ± 1.44	45.0 ± 4.7
**Age (yrs)**	65.5 ± 8.7	70.1 ± 10.3	68.7 ± 9.2	66.7 ± 8.9	65.0 ± 8.3	62.2 ± 7.8
**Sex**						
**Female N (%)**	4732 (61.1%)	402 (69.8%)	1178 (55.3%)	1313 (56.6%)	948 (64.5%)	891 (71.1%)
**Male N (%)**	3016 (38.9%)	174 (30.2%)	952 (44.7%)	1006 (43.4%)	522 (35.5%)	362 (28.9%)
**# of Comorbidities N (%)**						
**0**	6141 (79.3%)	460 (79.9%)	1762 (82.7%)	1848 (79.7%)	1152 (78.4%)	919 (73.3%)
**1**	1439 (18.6%)	101 (17.5%)	338 (15.9%)	419 (18.1%)	286 (19.4%)	295 (23.5%)
**2**	163 (2.1%)	15 (2.6%)	29 (1.3%)	48 (2.1%)	32 (2.2%)	39 (3.2%)
**3**	5 (0.1%)	0 (0.0%)	1 (0.1%)	4 (0.2%)	0 (0.0%)	0 (0.0%)

^a^Continuous variables are presented as the mean and the standard deviation. Categorical variables are presented as the number with the percentage in parentheses

Comorbidities included diabetes, moderate or severe mental health issues, cardiac disease, pulmonary disease, circulatory/clotting disorder, dementia, renal failure, cerebrovascular disease, and moderate or severe liver disease as recorded in the database

Patients in higher BMI groups were younger (*p* < .0001) compared to patients with normal BMI. The mean age at surgery ranged from 70.1 ± 10.3 in the BMI normal group to 62.2 ± 7.8 years in the BMI class III group. Patients in higher BMI groups were also more likely to be female, have a higher number of comorbidities, and, on average, have higher 30-day readmission. Patients’ self-reported preoperative measures of WOMAC total score, pain, stiffness, function, as well as EQ5D, on average (unadjusted), were 54.2, 53.4, 56.8, 54.1, and 0.51, respectively. These baseline WOMAC scores indicate that the patients were in moderate to severe condition.

### BMI groups and WOMAC subscales

At baseline, the adjusted means for WOMAC total score, pain, physical function, and stiffness were similar across different BMI groups (Supplementary Table 3.S). The adjusted mean at baseline for WOMAC total scores, pain, physical function, and stiffness were 56.3, 55.3, 56.6, and 57.2, respectively.

Adjusted mean changes (baseline to 3 months follow-up, 3 to 12 months follow-up, and baseline to 12 months follow-up) by BMI group for each of the WOMAC subscales are presented in Table 2. From baseline to 3 months, the adjusted mean change (improvement) in all WOMAC subscales were significant (adjusted *p* < .0001) for all BMI groups. From baseline to 3 months, all BMI groups experienced similar improvement (mean change) in WOMAC total, function, and stiffness. However, patients in the BMI class II and class III groups compared to BMI normal experienced significantly (*p* < .01) greater improvement (reduction) in pain from baseline to 3 months postoperatively.

**Table 2:** Mean changes in WOMAC and EQ5D by BMI group: Results from Mixed Effect Model^a^

Outcomes	Pre to 3 months	3 to 12 months	Pre to 12 months
	Mean change (95% C.I)	Mean change (95% C.I)	Mean change (95% C.I)
**Total Score†**			
Normal	−25.9(− 28.3, − 23.5)	−3.1(−6.8, 0.6)	−29.0(− 32.5, − 25.5)
Overweight	− 27.5(− 28.7, − 26.3)	− 3.5(− 5.3, 1.6)	−31.0(− 32.8, − 29.3)
Obese I	−28.5(− 29.7, − 27.3)	−3.3(− 5.1, − 1.5)	− 31.8(− 33.6, − 30.0)
Obese II	−28.9(− 30.5, − 27.3)	−2.1(− 4.5, 0.3)	− 31.0(− 33.2, − 28.8)
Obese III	− 29.1(− 30.7, − 27.5)	−2.0(− 4.5, 0.5)	−31.1(− 33.3, − 28.6)
Mean improvement = − 30.8			
**Pain†**			
Normal	−24.4(− 26.9, − 21.9)	−5.3(− 9.2, − 1.4)	− 29.8(− 33.3, − 26.3)
Overweight	−26.5(− 27.9, − 25.1)	−6.0(− 8.0, − 4.1)	− 32.6(− 34.8, − 30.8)
Obese I	−27.6(− 28.8, − 26.4)	− 5.2(− 7.2, − 3.2)	−32.8(− 34.6, − 31.0)
Obese II	**− 28.9(− 30.5, − 27.3)**	− 3.0(− 5.4, − 0.5)	− 31.9(− 34.2, − 29.7)
Obese III	**− 29.5(− 31.3, − 27.7)**	−3.2(− 5.7, − 0.7)	− 32.7(− 35.2, − 30.2)
Mean improvement = − 32.0			
**Function†**			
Normal	−26.8(− 29.2, − 24.4)	−2.1(− 5.8, 1.6)	− 28.9(− 32.2, − 25.6)
Overweight	−28.8(− 30.0, − 27.6)	−2.2(− 4.0, 0.3)	−31.0(− 23.7, − 29.3)
Obese I	−29.7(− 30.9, − 28.5)	−2.1(− 3.9, − 0.3)	− 31.9(− 33.7, − 30.1)
Obese II	−30.1(− 31.7, − 28.5)	−1.2(− 3.5, 1.1)	− 31.3(− 33.4, − 29.1)
Obese III	−30.3(− 31.9, − 28.7)	−0.9(− 3.4, 1.7)	−31.1(− 33.4, − 28.7)
Mean improvement = − 30.8			
**Stiffness†**			
Normal	−19.8(− 22.5, − 17.1)	− 7.6(− 11.9, − 3.3)	− 27.4(− 31.5, − 23.3)
Overweight	−21.8(− 23.2,20.4)	− 6.8(− 8.9, − 4.6)	− 28.6(− 30.6, − 26.6)
Obese I	−22.2(− 23.6, − 20.8)	−7.5(− 9.7, − 5.3)	− 29.8(− 31.8,− 27.8)
Obese II	−23.7(− 25.5, − 21.9)	−4.2(− 6.7, − 1.7)	− 27.9(− 30.3, − 25.5)
Obese III	−23.0(− 25.0, − 21.0)	− 5.5(− 8.4, − 2.6)	− 28.5(− 31.2, − 25.7)
Mean improvement = − 28.4			
**EQ5D**Ψ			
Normal	0.23 (0.19, 0.27)	0.01(−0.07, 0.09)	0.23 (0.17, 0.29)
Overweight	0.25 (0.23, 0.27)	0.01(−0.05, 0.07)	0.26 (0.22, 0.30)
Obese I	0.25 (0.23, 0.27)	0.01(−0.03, 0.05)	0.27 (0.23, 0.31)
Obese II	0.29 (0.27, 0.31)	0.01(−0.05, 0.03)	0.28 (0.24, 0.32)
Obese III	0.30 (0.27, 0.32)	0.01(−0.05, 0.03)	0.29 (0.25, 0.33)
Mean improvement = 0.27			

^a^The independent variables included in the model were BMI, time, BMI*time, age, sex, number of comorbidities, length of surgery, year of surgery, and geographic zone of service; Significant (*p* < .05) mean changes are **bolded and** show significance within a column**.** Negative mean changes for WOMAC scores (total, pain, function, and stiffness) indicate improvement. **†** Scale of 0 to 100, with 100 being the worst. Ψ1.00 indicating full health and 0 representing death

Patients in all BMI groups continued to experience significant (adjusted *p < .*0001) improvement in all WOMAC subscales in the time interval of 3 to 12 months following TKA, though the magnitudes were smaller compared to the improvement from baseline to 3 months follow-up. From 3 to 12 months follow-up, all BMI groups experienced similar improvement (mean change) in all WOMAC subscales.

From baseline to 12 months following TKA, the adjusted mean changes (improvement) in all WOMAC subscales were significant (p < .0001) for all BMI groups, and all groups experienced similar magnitude of improvement. On average, the improvement for WOMAC total score as well as pain, function, and stiffness were − 30.8, − 32.0, − 30.8, and − 28.4 points, respectively (Table 2).

### BMI groups and EQ5D

At baseline, the adjusted mean for the EQ5D index was not significantly different across BMI groups (Supplementary Table 3.S). The adjusted mean for the EQ5D index was 0.44 across all BMI groups. The adjusted mean changes (baseline to 3 months, 3 to 12 months, and baseline to 12 months) by BMI group for the EQ5D index are presented in Table 2. From baseline to 3 months, the adjusted mean change (improvement) in the EQ5D index was significant (adjusted *p* < .0001) in all BMI groups, and all groups experienced similar improvement. In the time interval of 3 to 12 months follow-up, the improvement in the EQ5D index almost plateaued. From baseline to 12 months follow-up, the adjusted mean changes (improvement) in EQ5D were significant (p < .0001) for all BMI groups, and all groups experienced similar improvement. On average, the improvement for EQ5D was 0.27.

## Discussion

In the present study, we evaluated the association between BMI groups, categorized according to WHO classification, with WOMAC and EQ5D preoperatively (baseline) and at different time intervals. There were no significant differences in self-reported preoperative pain, function, stiffness, or quality of life measures across all BMI groups. Our results indicate that by the end of 12 months follow-up all patients, regardless of their BMI, had improvement in pain, stiffness, physical function, and quality of life, and the magnitude of improvement was similar across all BMI groups.

The evidence of the impact of BMI on TKA outcomes in terms of PROMs is conflicting. Some studies suggest there is an association between obesity and pain, functional recovery, and quality of life following TKA [11–13, 39, 40], while others suggest no association [15, 41–43]. The variation in findings may be related to differences in the overall health status of the cohorts, use of different cut-offs for BMI, lack of control for confounding factors such as age and sex, and the small sample size [20, 21].

Recently, a study in the U.S. population by Collins and colleagues [17] examined the association between BMI groups, using the recommended WHO classification, and WOMAC subscale of function. They demonstrated that subjects with higher BMI had worse preoperative WOMAC pain and function than patients with normal BMI. Studies by Baker et al. in the U.K. demonstrated that patients in higher BMI groups (assessed in groups of BMI of < 25, 25 to 39.9, and ≥ 40 kg/m^2^) also had significantly (*p* < *.*01) worse preoperative WOMAC total and EQ5D scores (*p* < .001) [15, 43] than patients with normal BMI. We also observed that patients with higher BMI had poorer function, pain, and total scores at baseline, but these differences between BMI groups were not significant. The average baseline scores in our study compared to the U.S. population [17] were higher for pain (53.4 vs. 40.8) and function (54.1 vs. 42.5); whereas, the average baseline WOMAC total score was lower in our study compared to the U.K. population (54.2 vs. 63.2) [43]. Indeed, our patients had worse preoperative pain and function compared to U.S. patients, but better preoperative health status compared to the U.K patients. This discrepancy may be due to different health care systems in the U.S. and U.K. and Canada where different indication criteria and algorithms/cut-offs are used to guide the appropriateness of TKA [44–46].

Collins and colleagues [17] reported that patients in higher BMI groups experienced greater (*p* < .001) improvement in pain and function from baseline to 3 months after TKA compared to the lower BMI groups, but all groups had similar levels of pain and function at 24 months. We observed a greater improvement in pain from baseline to 3 months postoperatively in patients with higher BMI. However, all BMI groups attained similar level of pain reduction at 12 months after surgery. Baker et al. [43] reported that the average change for WOMAC total score from baseline to 12-month following TKA was similar across different BMI groups. Giesinger et al. [18] used WHO classification to categorize patients, with the Oxford Knee Score (OKS) used to measure self-reported pain and function, and the EQ-5D-3L used to measure general health status. They found no influence of BMI on postoperative self-reported pain, function, or general health scores. Our results were in line with the previous studies demonstrating that all patients received the same benefit from TKA regardless of their BMI [15, 17, 18, 43], and most of the improvement occurred by 3 months postoperatively [17].

Similar to other studies [15, 17, 18, 43], at the end of the study period, all BMI groups experienced statistically significant and clinically meaningful improvement in pain, function, stiffness, and total WOMAC score as well as EQ5D index. Despite substantial improvement in pain and function after TKA across all BMI groups, at the end of 12 months after surgery, our patients experienced worse pain and function than patients in Collins et al. study [17]. This may be explained by the worse baseline pain and function of our participants compared to Collins et al. study, as preoperative health status affects the postoperative outcomes [47].

We examined the PROMs preoperatively, pre- to 3 months postoperatively, as well as 3 to 12 months after TKA, and the findings of our study offer insight into the association between different grades of obesity and PROMs in Albertans following TKA. Although studies demonstrated that obesity places patients at increased risk of adverse events after TKA [48, 49], in the current study, patients in higher BMI groups experienced improvements in PROMs similar to those of patients in the normal BMI group. An important takeaway is that healthcare providers and surgeons should consider performing TKA in patients with higher BMI in the absence of weight loss or willingness to lose weight as studies show that delaying the surgery will lead to worse outcomes as well as higher anxiety and depression in patients [50].

### Strengths and limitations

The strength of this study includes a large sample of patients (*N* = 7714) with WOMAC total score and 3 subscales recorded at baseline and 3 and/or 12 months postoperatively. We have also categorized patients into 5 groups based on the WHO classification of BMI, which helped us to evaluate a clear relationship between each of the BMI groups with TKA outcomes. BMI records in our dataset were not self-reported, which provides more reliable results. Our analysis also had limitations inherent to retrospective studies. WOMAC was used to measure lower extremity physical function, which has been reported to have limited ability to accurately predict change in function [51]. There are also other common health metrics including Patient-Reported outcome measurement Information System (PROMIS) used for knee conditions. However, their results are not condition-specific and can be mostly used to compare with population norms [52]. Studies also demonstrated no differences in the internal and external responsiveness of these measures with other health measures such as EQ5D used in TKA setting. In the current study, WOMAC and EQ5D has been used as a primary outcome, since ABJHI collects PROMs as part of routine clinical care measurements, and these are widely used PROMs in knee arthroplasty settings. There was a temporal-based improvement in BMI and PROM data recording process. Individuals who did not have weight and height records were excluded from the study, though there were no significant differences in patient-reported outcomes between the cohort that was excluded and those included in the cohort studied (Supplementary: Table 1.S and Table 2.S). In this study, BMI has been used as a measure of obesity, however, BMI does not provide us with information about the body’s fat distribution as well as body composition [53]. Further studies using methods such waist to hip ratios or sophisticated methods such as dual-energy X-ray absorptiometry (DEXA) are recommended to evaluate the association between fat distribution and body composition with TKA outcomes in patients who have undergone surgery. Patients who had baseline and at least 1 follow-up visit (postoperative month 3 or 12) for WOMAC and EQ5D questionnaires were included in the study. Given that not all patients had both EQ5D and WOMAC measurements recorded, two separate datasets (WOMAC dataset and EQ5D datasets) were prepared for this study. Then, WOMAC scores were not adjusted for the baseline measures of EQ5D and vice versa. Patients with missing follow-up questionnaires were also included in the analysis as linear mixed effect models allow for missing data and are robust to determining estimates in presence of missing data [54]. In this study, only data from 2012 to 2016 were acquired from ABJHI and included in the analysis. However, information from these years could provide some insights into answering our question of interest.

## Conclusions

Overall, we found that participants across all BMI groups achieved a similar benefit with respect to patients’ self-reported outcomes of WOMAC scores (pain, function, stiffness, and total score) and EQ5D by the end of 12 months following TKA. Patients in the obese class II and III groups achieved more benefits (although not clinically meaningful in terms of pain outcome compared to normal groups by 3 months), but all BMI groups were able to attain the same benefit by the end of 12 months following TKA. The majority of improvement for all WOMAC subscales and the EQ5D occurred by 3 months after surgery. These results may help health care providers to discuss expectations regarding the TKA recovery in terms of pain, function, and quality of life improvements with their TKA candidates.

## Supplementary Information

**Additional file 1.**

## Data Availability

All data supporting the results of this study are stored at ABJHI repository www.albertaboneandjoint.com. Data are not publicly available, and permission to extract data is obtained from the registry holder.

## Abbreviations

OA: Osteoarthritis
TKA: Total Knee Arthroplasty
BMI: Body Mass Index
ABJHI: Alberta Bone and Joint Health Institute
WHO: World Health Organization
CI: Confidence Intervals
WOMAC: Western Ontario and McMaster Universities Osteoarthritis Index
EQ5D: EuroQol-5D
PROMs: Patient-reported Outcome Measures
MCID: Minimal Clinically Important Difference

## References

[1] Bombardier C, Hawker G, Mosher D (2016). Impact on arthrisis in Canada_Today and over the next 30 years. Arthritis Alliance of Canada.

[2] Cross M, Smith E, Hoy D, Nolte S, Ackerman I, Fransen M, Bridgett L, Williams S, Guillemin F, Hill CL (2014). The global burden of hip and knee osteoarthritis: estimates from the global burden of disease 2010 study. Ann Rheum Dis.

[3] Adie S, Harris I, Chuan A, Lewis P, Naylor JM (2019). Selecting and optimising patients for total knee arthroplasty. Med J Aust.

[4] Chesworth BM, Mahomed NN, Bourne RB, Davis AM, Group OS (2008). Willingness to go through surgery again validated the WOMAC clinically important difference from THR/TKR surgery. J Clin Epidemiol.

[5] Bachmeier CJ, March LM, Cross MJ, Lapsley HM, Tribe KL, Courtenay BG, Brooks PM, Arthritis C, Outcome Project G (2001). A comparison of outcomes in osteoarthritis patients undergoing total hip and knee replacement surgery. Osteoarthr Cartil.

[6] Lee R, Kean WF (2012). Obesity and knee osteoarthritis. Inflammopharmacology.

[7] Werner BC, Evans CL, Carothers JT, Browne JA (2015). Primary Total knee arthroplasty in super-obese patients: dramatically higher postoperative complication rates even compared to revision surgery. J Arthroplast.

[8] Della Valle CJ (2017). Body mass index and outcomes of TKA: insight for counseling patients: commentary on an article by Jamie E. Collins, PhD, et al.: "effect of obesity on pain and functional recovery following Total knee arthroplasty". J Bone Joint Surg Am.

[9] van der Merwe JM, Mastel MS. Controversial topics in Total knee arthroplasty: a 5-year update (part 1). J Am Acad Orthop Surg Glob Res Rev. 2020;4(1):e1900047. 10.5435/JAAOSGlobal-D-19-00047 PMC7028773 32672726

[10] Papakostidou I, Dailiana ZH, Papapolychroniou T, Liaropoulos L, Zintzaras E, Karachalios TS, Malizos KN (2012). Factors affecting the quality of life after total knee arthroplasties: a prospective study. BMC Musculoskelet Disord.

[11] Stevens-Lapsley JE, Petterson SC, Mizner RL, Snyder-Mackler L (2010). Impact of body mass index on functional performance after total knee arthroplasty. J Arthroplast.

[12] Vincent HK, Vincent KR (2008). Obesity and inpatient rehabilitation outcomes following knee arthroplasty: a multicenter study. Obesity (Silver Spring).

[13] Pua YH, Seah FJ, Seet FJ, Tan JW, Liaw JS, Chong HC (2015). Sex differences and impact of body mass index on the time course of knee range of motion, knee strength, and gait speed after Total knee arthroplasty. Arthritis Care Res.

[14] Chen JY, Lo NN, Chong HC, Bin Abd Razak HR, Pang HN, Tay DK, Chia SL, Yeo SJ (2016). The influence of body mass index on functional outcome and quality of life after total knee arthroplasty. Bone Joint J.

[15] Baker P, Petheram T, Jameson S, Reed M, Gregg P, Deehan D (2012). The association between body mass index and the outcomes of total knee arthroplasty. J Bone Joint Surg Am.

[16] Keeney BJ, Austin DC, Jevsevar DS (2019). Preoperative weight loss for morbidly obese patients undergoing Total knee arthroplasty: determining the necessary amount. J Bone Joint Surg Am.

[17] Collins JE, Donnell-Fink LA, Yang HY, Usiskin IM, Lape EC, Wright J, Katz JN, Losina E (2017). Effect of obesity on pain and functional recovery following Total knee arthroplasty. J Bone Joint Surg Am.

[18] Giesinger JM, Loth FL, MacDonald DJ, Giesinger K, Patton JT, Simpson A, Howie CR, Hamilton DF (2018). Patient-reported outcome metrics following total knee arthroplasty are influenced differently by patients' body mass index. Knee Surg Sports Traumatol Arthrosc.

[19] Jester R, Rodney A (2021). The relationship between obesity and primary Total knee replacement: a scoping review of the literature. Int J Orthop Trauma Nurs.

[20] Pozzobon D, Ferreira PH, Blyth FM, Machado GC, Ferreira ML (2018). Can obesity and physical activity predict outcomes of elective knee or hip surgery due to osteoarthritis? A meta-analysis of cohort studies. BMJ Open.

[21] Workgroup of the American Association of H, knee surgeons evidence based C (2013). Obesity and total joint arthroplasty: a literature based review. J Arthroplast.

[22] Conner-Spady BL, Marshall DA, Bohm E, Dunbar MJ, Loucks L, Al Khudairy A, Noseworthy TW (2015). Reliability and validity of the EQ-5D-5L compared to the EQ-5D-3L in patients with osteoarthritis referred for hip and knee replacement. Qual Life Res.

[23] Bellamy N, Buchanan WW, Goldsmith CH, Campbell J, Stitt LW (1988). Validation study of WOMAC: a health status instrument for measuring clinically important patient relevant outcomes to antirheumatic drug therapy in patients with osteoarthritis of the hip or knee. J Rheumatol.

[24] Bellamy N (1995). Outcome measurement in osteoarthritis clinical trials. J Rheumatol Suppl.

[25] Obesity: preventing and managing the global epidemic. Report of a WHO consultation. World Health Organ Tech Rep Ser. 2000;894:i-xii, 1-253. 11234459

[26] Copsey B, Thompson JY, Vadher K, Ali U, Dutton SJ, Fitzpatrick R, Lamb SE, Cook JA (2019). Problems persist in reporting of methods and results for the WOMAC measure in hip and knee osteoarthritis trials. Qual Life Res.

[27] Singh J, Sloan JA, Johanson NA (2010). Challenges with health-related quality of life assessment in arthroplasty patients: problems and solutions. J Am Acad Orthop Surg.

[28] Bellamy N (2000). WOMAC osteoarthritis index user guide IV.

[29] Terwee CB, Roorda LD, Knol DL, De Boer MR, De Vet HC (2009). Linking measurement error to minimal important change of patient-reported outcomes. J Clin Epidemiol.

[30] Impellizzeri FM, Mannion AF, Leunig M, Bizzini M, Naal FD (2011). Comparison of the reliability, responsiveness, and construct validity of 4 different questionnaires for evaluating outcomes after total knee arthroplasty. J Arthroplast.

[31] Clement ND, Bardgett M, Weir D, Holland J, Gerrand C, Deehan DJ (2018). What is the minimum clinically important difference for the WOMAC index after TKA?. Clin Orthop Relat Res.

[32] Angst F, Benz T, Lehmann S, Aeschlimann A, Angst J (2018). Multidimensional minimal clinically important differences in knee osteoarthritis after comprehensive rehabilitation: a prospective evaluation from the bad Zurzach osteoarthritis study. RMD Open.

[33] Information CIfH (2015). PROMs forum proceedings.

[34] Jin X, Al Sayah F, Ohinmaa A, Marshall DA, Smith C, Johnson JA (2019). The EQ-5D-5L is superior to the -3L version in measuring health-related quality of life in patients awaiting THA or TKA. Clin Orthop Relat Res.

[35] Janssen MF, Pickard AS, Golicki D, Gudex C, Niewada M, Scalone L, Swinburn P, Busschbach J (2013). Measurement properties of the EQ-5D-5L compared to the EQ-5D-3L across eight patient groups: a multi-country study. Qual Life Res.

[36] EuroQol G (1990). EuroQol--a new facility for the measurement of health-related quality of life. Health Policy.

[37] Conner-Spady BL, Marshall DA, Bohm E, Dunbar MJ, Noseworthy TW (2018). Comparing the validity and responsiveness of the EQ-5D-5L to the Oxford hip and knee scores and SF-12 in osteoarthritis patients 1 year following total joint replacement. Qual Life Res.

[38] Fox J. Regression diagnostics: quantitative applications in the social sciences. Series no. 79. Thousands Oaks (CA): Sage Publications; 1991.

[39] Naylor JM, Harmer AR, Heard RC (2008). Severe other joint disease and obesity independently influence recovery after joint replacement surgery: an observational study. Aust J Physiother.

[40] Deshmukh RG, Hayes JH, Pinder IM (2002). Does body weight influence outcome after total knee arthroplasty? A 1-year analysis. J Arthroplast.

[41] Singh JA, Gabriel SE, Lewallen DG (2011). Higher body mass index is not associated with worse pain outcomes after primary or revision total knee arthroplasty. J Arthroplast.

[42] Dewan A, Bertolusso R, Karastinos A, Conditt M, Noble PC, Parsley BS (2009). Implant durability and knee function after total knee arthroplasty in the morbidly obese patient. J Arthroplast.

[43] Baker P, Muthumayandi K, Gerrand C, Kleim B, Bettinson K, Deehan D (2013). Influence of body mass index (BMI) on functional improvements at 3 years following total knee replacement: a retrospective cohort study. PLoS One.

[44] Riddle DL, Jiranek WA, Hayes CW (2014). Use of a validated algorithm to judge the appropriateness of total knee arthroplasty in the United States: a multicenter longitudinal cohort study. Arthritis Rheum.

[45] Gademan MG, Hofstede SN, Vliet Vlieland TP, Nelissen RG, Marang-van de Mheen PJ (2016). Indication criteria for total hip or knee arthroplasty in osteoarthritis: a state-of-the-science overview. BMC Musculoskelet Disord.

[46] Hawker G, Bohm ER, Conner-Spady B, De Coster C, Dunbar M, Hennigar A (2015). Perspectives of Canadian stakeholders on criteria for appropriateness for Total joint arthroplasty in patients with hip and knee osteoarthritis. Arthritis Rheum..

[47] Hofstede SN, Gademan MGJ, Stijnen T, Nelissen R, Marang-van de Mheen PJ, group A-Os (2018). The influence of preoperative determinants on quality of life, functioning and pain after total knee and hip replacement: a pooled analysis of Dutch cohorts. BMC Musculoskelet Disord.

[48] George J, Piuzzi NS, Ng M, Sodhi N, Khlopas AA, Mont MA (2018). Association between body mass index and thirty-day complications after Total knee arthroplasty. J Arthroplast.

[49] Sloan M, Sheth N, Lee GC (2019). Is obesity associated with increased risk of deep vein thrombosis or pulmonary embolism after hip and knee arthroplasty? A large database study. Clin Orthop Relat Res.

[50] Conner-Spady B, Estey A, Arnett G, Ness K, McGurran J, Bear R, Noseworthy T, Steering Committee of the Western Canada Waiting List P (2004). Prioritization of patients on waiting lists for hip and knee replacement: validation of a priority criteria tool. Int J Technol Assess Health Care.

[51] Stratford P, Kennedy D, Clarke H (2018). Confounding pain and function: the WOMAC's failure to accurately predict lower extremity function. Arthroplast Today.

[52] Cella D, Hahn EA, Jensen SE, Butt Z, Nowinski CJ, Rothrock N, Lohr KN (2015). Patient-reported outcomes in performance Measurement.

[53] Shah NR, Braverman ER (2012). Measuring adiposity in patients: the utility of body mass index (BMI), percent body fat, and leptin. PLoS One.

[54] Ibrahim JG, Molenberghs G (2009). Missing data methods in longitudinal studies: a review. Test (Madr).

