# Supplementary material for: Does obesity affect patient-reported outcomes following total knee arthroplasty?
Source: BMC Musculoskelet Disord. 2022 Jan 17;23:55. doi: 10.1186/s12891-022-04997-4 (PMC8764810; doi:10.1186/s12891-022-04997-4)
Supplement: Supplementary file 1 — Additional file 1. [file 12891_2022_4997_MOESM1_ESM.docx]

### **Supplementary Materials**

| **Table 1.S.** Comparison of patient characteristics between those included (BMI data available) vs. excluded (no BMI data available) in the analysis**^£^** | | | | | |
| --- | --- | --- | --- | --- | --- |
|  | **Included (N=7714)** |  | **Excluded(N=5650)** |  | **P-Value** |
| **Age (yrs)** | 65.5±1.1 |  | 66.5±1.1 |  | 0.34 |
| **Sex** | | | | | |
| **Female N (%)**  **Male N (%)** | 4732(61.1)  3016(38.9) |  | 3016(58.8)  2330(41.2) |  | 0.51  0.51 |
| **# of Comorbidities** | | | | | |
| **0**  **1**  **2**  **3** | 6141(79.3)  1439(18.6)  163(2.1)  5(0.1) |  | 4457(78.9)  1046(18.5)  141(2.5)  5(0.1) |  | 0.92  0.96  0.56  0.44 |
| **^£^**Continuous variables are presented as the mean and the standard error of mean. Categorical variables are presented as the number, with the percentage in parentheses. | | | | | |

| **Table 2.S.** Adjusted mean comparison between those included (BMI data available) vs. excluded (no BMI data available) cohorts for Western Ontario and McMaster Universities Osteoarthritis Index (WOMAC) subscales and EuroQol-5D (EQ5D) ^£^ | | | | | | | | | |
| --- | --- | --- | --- | --- | --- | --- | --- | --- | --- |
|  |  | | **Included cohort**  **(With BMI records)** | | **Excluded cohort**  **(Without BMI records)** | |  |  | |
| **Outcomes** | **Time** | **N** | | **Mean** | **N** | **Mean** | **SEM** | **P-Value** | |
| **Total Score** | | | | | | | | |  |
|  | **Pre** | 7714 | | 55.6 | 5650 | 54.8 | 0.53 | 0.13 | |
|  | **Post 3 months** | 3846 | | 26.3 | 2302 | 25.7 | 0.73 | 0.35 | |
|  | **Post 12 months** | 1663 | | 25.3 | 1074 | 23.2 | 1.45 | 0.10 | |
| **Pain** | | | | | | | | |  |
|  | **Pre** | 7714 | | 54.6 | 5650 | 53.6 | 0.56 | 0.10 | |
|  | **Post 3 months** | 3846 | | 23.9 | 2302 | 23.2 | 0.70 | 0.15 | |
|  | **Post 12 months** | 1663 | | 24.6 | 1074 | 21.6 | 1.49 | 0.06 | |
| **Function** | | | | | | | | |  |
|  | **Pre** | 7714 | | 56.0 | 5650 | 55.2 | 0.55 | 0.16 | |
|  | **Post 3 months** | 3846 | | 28.0 | 2302 | 28.1 | 0.75 | 0.84 | |
|  | **Post 12 months** | 1663 | | 26.0 | 1074 | 22.6 | 1.41 | 0.07 | |
| **Stiffness** | | | | | | | | |  |
|  | **Pre** | 7714 | | 55.0 | 5650 | 54.3 | 0.61 | 0.26 | |
|  | **Post 3 months** | 3846 | | 33.5 | 2302 | 32.2 | 0.85 | 0.10 | |
|  | **Post 12 months** | 1663 | | 24.2 | 1074 | 22.5 | 1.53 | 0.27 | |
| **EQ5D** | | | | | | | | |  |
|  | **Pre** | 3848 | | 0.45 | 2763 | 0.47 | 0.009 | 0.04 | |
|  | **Post 3 months** | 1579 | | 0.76 | 1274 | 0.75 | 0.009 | 0.69 | |
|  | **Post 12 months** | 534 | | 0.68 | 435 | 0.67 | 0.02 | 0.75 | |
| **^£^** Adjusted means and standard error of mean differences were calculated from linear mixed model adjusted for age, sex, number of comorbidities, and zone of service. | | | | | | | | | |

| **Table 3.S.** Least square means of pain, physical function, stiffness, and total score at preoperative and 3- and 12- months following surgery for different body mass index groups | | | | |
| --- | --- | --- | --- | --- |
| **Outcomes** | | **Baseline** | **3 months** | **12 months** |
|  |  | **Mean (95% C.I)** | **Mean (95% C.I)** | **Mean (95% C.I)** |
| **Total Score†** | | | | |
|  | **Normal** | 54.9(50.8, 59.0) | 28.9(24.5, 33.3) | 26.0(20.9, 31.1) |
|  | **Overweight** | 55.4(51.5, 59.3) | 27.9(23.9, 31.9) | 24.3(20.2, 28.5) |
|  | **Obese I** | 56.7(52.8, 60.5) | 28.2(24.2, 32.1) | 24.8(20.7, 28.9) |
|  | **Obese II** | 56.9(52.9, 61.6) | 28.0(23.9, 32.0) | 25.6(21.3, 29.9) |
|  | **Obese III** | 57.6(53.7, 61.6) | 28.7(24.6, 32.8) | 26.6(22.2, 31.1) |
| **Pain†** | | | | |
|  | **Normal** | 54.1(49.8, 58.5) | 29.6(25.0, 34.2) | 24.4(19.1, 29.7) |
|  | **Overweight** | 54.9(50.8, 59.1) | 28.4(24.2, 32.6) | 22.3(17.9, 26.7) |
|  | **Obese I** | 55.8(51.7, 59.9) | 28.2(24.0, 32.4) | 23.0(18.7, 27.4) |
|  | **Obese II** | 55.3(51.1, 59.5) | 26.4(22.1, 30.7) | 23.4(18.8, 28.0) |
|  | **Obese III** | 56.2(52.0, 60.4) | 26.8(22.5, 31.1) | 23.2(18.6, 27.9) |
| **Function†** | | | | |
|  | **Normal** | 55.0(50.7, 59.3) | 28.0(23.5, 32.5) | 26.1(21.0, 31.2) |
|  | **Overweight** | 55.5(51.4, 59.3) | 26.7(22.6, 30.8) | 24.5(20.2, 28.7) |
|  | **Obese I** | 56.9(52.8, 60.9) | 27.1(23.0, 31.2) | 25.0(20.8, 29.2) |
|  | **Obese II** | 57.3(53.3, 61.4) | 27.2(23.0, 31.4) | 26.0(21.6, 30.5) |
|  | **Obese III** | 58.2(54.1, 62.3) | 28.0(23.8, 32.2) | 27.1(22.6, 31.7) |
| **Stiffness†** | | | | |
|  | **Normal** | 57.0(52.4, 61.6) | 37.1(32.2, 42.0) | 29.6(23.8, 35.3) |
|  | **Overweight** | 56.7(52.4, 61.1) | 34.9(30.5, 39.3) | 28.1(23.5, 32.8) |
|  | **Obese I** | 57.5(53.2, 61.8) | 35.2(30.8, 39.7) | 27.7(23.1, 32.8) |
|  | **Obese II** | 57.5(53.1, 61.9) | 33.8(29.2, 38.3) | 29.4(24.5, 34.2) |
|  | **Obese III** | 57.2(52.8, 61.6) | 34.3(29.8, 38.9) | 28.9(23.9, 33.8) |
| **EQ5D**Ψ | | | | |
|  | **Normal** | 0.46(0.37, 0.55) | 0.69(0.6, 0.79) | 0.69(0.58, 0.80) |
|  | **Overweight** | 0.44(0.35, 0.53) | 0.70(0.61, 0.79) | 0.70(0.61, 0.80) |
|  | **Obese I** | 0.45(0.36, 0.54) | 0.70(0.61, 0.79) | 0.71(0.62, 0.81) |
|  | **Obese II** | 0.42(0.33, 0.51) | 0.70(0.61, 0.80) | 0.70(0.60, 0.80) |
|  | **Obese III** | 0.41(0.32, 0.50) | 0.71(0.62, 0.80) | 0.72(0.62, 0.82) |
| **†** Scale of 0 to 100, with 100 being the worst. Ψ1.00 indicating full health and 0 representing death. | | | | |

| **Table 4.S.** Regression coefficient (standard error) of model parameters for different dependent variables | | | | | |
| --- | --- | --- | --- | --- | --- |
| **Model parameters** |  | **Dependent variable** | | | |
|  |  | **Total score**  **β(SE)** | **Pain**  **β(SE)** | **Stiffness**  **β(SE)** | **Function**  **β(SE)** |
| **(Intercept)** |  | 61.8(2.1) | 70.4(2.2) | 74.7(2.3) | 57.8(2.2) |
| **Length of surgery** |  | -0.006 | -0.015 | -0.005 | -0.003 |
| **BMI groups** |  |  |  |  |  |
| BMI2 (Overweight) |  | 0.48(0.9) | 0.8(0.9) | -0.2(1.0) | 0.5(0.9) |
| BMI3 (Obese Class I) |  | 1.7(0.9) | 1.7(0.9) | 0.5(1.0) | 1.9(0.9) |
| BMI4 (Obese Class II) |  | 1.98(0.9) | 1.2(1.0) | 0.5(1.1) | 2.4(1.0) |
| BMI5 (Obese Class III) | | 2.8(0.9) | 2.2(1.0) | 0.3(1.1) | 3.2(1.0) |
| **Time** |  |  |  |  |  |
| Time2 (post 3 months) |  | -26.0(1.2) | -24.6(1.3) | -19.9(1.4) | -27.0(1.2) |
| Time3 (post12months) |  | -28.9(1.8) | -29.8(1.8) | -27.4(2.1) | -28.9(1.8) |
| **Sex** |  |  |  |  |  |
| Male |  | -3.0(0.4) | -3.4(0.4) | -4.3(0.4) | -2.6(0.4) |
| **Age** |  | -0.1(0.0) | -0.2(0.0) | -0.3(0.0) | -0.1(0.0) |
| **Zone of Service** |  |  |  |  |  |
| Central |  | 1.0(0.5) | 1.8(0.6) | 1.1(0.6) | 0.9(0.6) |
| Edmonton |  | 2.0(0.4) | 1.7(0.4) | 1.9(0.5) | 2.3(0.4) |
| **Year of Surgery** |  |  |  |  |  |
| 2013 |  | -1.7(0.9) | -1.5(1.0) | -0.21(1.1) | -1.8(1.0) |
| 2014 |  | -1.2(0.9) | -1.2(1.0) | 0.02(1.1) | -1.3(1.0) |
| 2015 |  | -0.6(0.9) | -0.5(1.0) | 0.15(1.1) | -0.7(1.0) |
| 2016 |  | -2.0(1.0) | -2.3(1.1) | -1.9(1.6) | -2.1(1.1) |
| **# of Comorbidities** |  |  |  |  |  |
| 1 |  | 2.7(0.5) | 2.7(0.5) | 1.3(0.5) | 3.1(0.5) |
| 2 |  | 6.9(1.3) | 7.3(1.4) | 4.3(1.5) | 7.0(1.4) |
| 3 |  | 2.7(7.7) | 0.8(8.1) | -1.9(8.6) | 3.8(7.9) |
| **Time × BMI groups** |  |  |  |  |  |
| BMI 2: Time 2 |  | -1.5(1.3) | -2.0(1.4) | -1.9(1.6) | -1.8(1.4) |
| BMI 3: Time 2 |  | -2.5(1.3) | -3.0(1.4) | -2.4(1.6) | -2.7(1.4) |
| BMI 4: Time 2 |  | -2.9(1.4) | -4.4(1.5) | -3.8(1.7) | -3.1(1.4) |
| BMI 5: Time 2 |  | -3.0(1.4) | -4.8(1.6) | -3.0(1.7) | -3.2(1.5) |
| BMI 2: Time 3 |  | -2.1(2.0) | -2.8(2.1) | -1.2(2.3) | -2.1(2.0) |
| BMI 3: Time 3 |  | -2.9(2.0) | -3.0(2.0) | -2.4(2.3) | -3.0(2.0) |
| BMI 4: Time 3 |  | -2.4(2.1) | -2.1(2.2) | -0.7(2.4) | -2.4(2.1) |
| BMI 5: Time 3 |  | -2.1(2.1) | -3.1(2.2) | -0.9(2.5) | -2.2(2.1) |
| BMI:1 (Normal), Time: 1 (pre), Sex: Female, Year of Surgery: 2012, Zone: South, Comorbidities: Zero, and interaction of BMI 1by Time 1 were reference groups in multiple linear mixed effect models | | | | | |

**Results generated using raw data with range of 0-96 for total score, 0-68 for physical function, 0-20 for pain, and 0-8 for stiffness)**

a

b

c

d

**Figure 1.S.** **Adjusted mean for WOMAC total score (a), pain (b), physical function (c), and stiffness (d) by BMI group and time (baseline [preoperative] and 3, and 12 months post TKA).** Each line represents a BMI group normal weight (solid square; BMI≤24.99 kg/m^2^ BMI of <25 kg/m^2^), overweight (dashed diamond; 25≤BMI≤29.99 kg/m^2^), class-I obese (dashed triangle; 30≤BMI≤34.99 kg/m^2^), class-II obese (dashed square; 35≤BMI≤39.99 kg/m^2^), and class-III obese (solid circle; BMI≥40 kg/m^2^). Adjusted means were computed from a linear mixed effects model adjusting for age, sex, number of comorbidities, and zone of service. Least square mean values, which are used to generate the graphs are provided in the Supplement as Table 5.S.

| **Table 5.S.** Least square mean of Western Ontario and McMaster Universities Osteoarthritis Index (WOMAC) subscales using raw data at preoperative, 3- and 12-month postoperative | | | | |
| --- | --- | --- | --- | --- |
| **Outcomes** | | **Pre** | **3-month** | **12-month** |
|  |  | **Mean (95% C.I)** | **Mean (95% C.I)** | **Mean (95% C.I)** |
| **Total Score** (scale 0-96) | | | | |
|  | Normal | 52.7(56.7,40.3) | 27.8(32.0,22.1) | 24.9(29.8,21.2) |
|  | Overweight | 53.2(56.9,40.5) | 26.8(30.6,20.9) | 23.4(27.4,19.5) |
|  | Obese I | 54.4(58.1,41.4) | 27.0(30.8,21.2) | 23.8(27.8,19.9) |
|  | Obese II | 54.6(58.4,41.8) | 26.8(30.7,21.3) | 24.6(28.7,20.7) |
|  | Obese III | 55.3(59.1,42.4) | 27.5(31.4,21.9) | 25.6(2.2,21.5) |
| **Pain** (scale 0-20) | | | | |
|  | Normal | 10.8(10,11.7) | 5.9(5,6.9) | 4.9(3.8,5.9) |
|  | Overweight | 11.0(10.2,11.8) | 5.7(4.8,6.5) | 4.5(3.6,5.4) |
|  | Obese I | 11.2(10.3,12) | 5.7(4.8,6.5) | 4.6(3.7,5.5) |
|  | Obese II | 11.1(10.2,11.9) | 5.3(4.4,6.1) | 4.7(3.8,5.6) |
|  | Obese III | 11.2(10.4,12.1) | 5.4(4.5,6.2) | 4.7(3.7,5.6) |
| **Function** (scale 0-68) | | | | |
|  | Normal | 37.4(34.5,40.3) | 19.0(16.0,22.1) | 17.7(14.3,21.2) |
|  | Overweight | 37.7(35,40.5) | 18.1(15.3,20.9) | 16.6(13.7,19.5) |
|  | Obese I | 38.7(35.9,41.4) | 18.4(15.7,21.2) | 17.0(14.1,19.9) |
|  | Obese II | 39(36.2,41.8) | 18.5(15.7,21.3) | 17.7(14.7,20.7) |
|  | Obese III | 39.6(36.8,42.4) | 19.1(16.2,21.9) | 18.4(15.4,21.5) |
| **Stiffness** (scale 0-8) | | | | |
|  | Normal | 4.6(4.2,4.9) | 3.0(2.6,3.4) | 2.4(1.9,2.8) |
|  | Overweight | 4.5(4.2,4.9) | 2.8(2.4,3.2) | 2.3(1.9,2.6) |
|  | Obese I | 4.6(4.3,5.0) | 2.8(2.5,3.2) | 2.2(1.9,2.6) |
|  | Obese II | 4.6(4.3,5.0) | 2.7(2.3,3.1) | 2.4(2.0,2.7) |
|  | Obese III | 4.6(4.2,4.9) | 2.8(2.4,3.1) | 2.3(1.9,2.7) |

| **Table 6.S.** Mean Changes by BMI Group: Results from Mixed Effect Model Using raw data | | | | |
| --- | --- | --- | --- | --- |
| **Outcomes** | | **Pre to 3 M** | **3 to 12 months** | **Pre to 12 months** |
|  |  | **Mean change (95% C.I)** | **Mean change (95% C.I)** | **Mean change (95% C.I)** |
| **Total Score** (scale 0-96) | | | | |
|  | Normal | -25.0(-27.2, -22.7) | -2.8(-6.4, -0.8) | -27.8(-31.1, -24.4) |
|  | Overweight | -26.4(-27.6, -25.3) | -3.4(-5.2, -1.6) | -29.8(-31.5, -28.2) |
|  | Obese I | -27.4(-28.5, -26.2) | -3.2(-4.9, -1.5) | -31.0(-32.7, -29.3) |
|  | Obese II | -27.8(-29.2, -26.3) | -2.3(-4.5, -0.1) | -30.1(-32.1, -28.0) |
|  | Obese III | -27.8(-29.3, -26.3) | -2.0(-4.3, 0.4) | -29.8(-32.0, -27.5) |
| Mean improvement = -29.7 | | | | |
| **Pain** (scale 0-20) | | | | |
|  | Normal | -4.9(-5.4, -4.4) | -1.0(-1.8, -0.3) | -6.0(-6.7, -5.2) |
|  | Overweight | -5.3(-5.6, -5.0) | -1.2(-1.6, -0.8) | -6.5(-6.9, -6.2) |
|  | Obese I | -5.5(-5.8, -5.3) | -1.0(-1.4, -0.7) | -6.7(-7.1, -6.3) |
|  | Obese II | **-5.8(-6.1, -5.5)** | -0.6(-1.1, -0.1) | -6.4(-6.8, -5.9) |
|  | Obese III | **-5.9(-6.2, -5.5)** | -0.7(-1.2, -0.2) | -6.6(-7.1, -6.1) |
| Mean improvement = -6.4 | | | | |
| **Function** (scale 0-68) | | | | |
|  | Normal | -18.3(-20.0, -16.7) | -1.3(-3.8, 1.2) | -19.6(-22.0, -17.3) |
|  | Overweight | -19.6(-20.4, -18.8) | -1.5(-2.7, -0.3) | -21.1(-22.3, -19.9) |
|  | Obese I | -20.2(-21.0, -19.4) | -1.4(-2.7, -0.2) | -22.0(-23.3, -20.8) |
|  | Obese II | -20.5(-21.5, -19.5) | -0.8(-2.4, 0.7) | -21.3(-22.7, -19.8) |
|  | Obese III | -20.5(-21.6, -19.4) | -0.6(-2.3, 1.1) | -21.1(-22.7, -19.5) |
| Mean improvement = -21.0 | | | | |
| **Stiffness** (scale 0-8) | | | | |
|  | Normal | -1.6(-1.8, -1.4) | -0.6(-0.9, -0.3) | -2.2(-2.5, -1.9) |
|  | Overweight | -1.7(-1.9, -1.6) | -0.5(-0.7, -0.4) | -2.3(-2.4, -2.1) |
|  | Obese I | -1.8(-1.9, -1.7) | -0.6(-0.8, -0.4) | -2.3(-2.5, -2.2) |
|  | Obese II | -1.9(-2.0, -1.8) | -0.4(-0.6, -0.1) | -2.2(-2.4, -2.1) |
|  | Obese III | -1.8(-2.0, -1.7) | -0.4(-0.7, -0.2) | -2.3(-2.5, -2.1) |
| Mean improvement = -2.3 | | | | |
| ^£^Significant mean changes are bolded. Negative mean changes for WOMAC scores (total, pain, function, and stiffness) indicate improvement. | | | | |

| **Table 7.S** Unadjusted mean changes in WOMAC and EQ5D by BMI group | | | | |
| --- | --- | --- | --- | --- |
| **Outcomes** | | **Pre to 3 months** | **3 to 12 months** | **Pre to 12 months** |
|  |  | **Mean change (95% C.I)** | **Mean change (95% C.I)** | **Mean change (95% C.I)** |
| **Total Score†** | | | | |
|  | Normal | -26.3(-28.7, -23.9) | -2.8(, -6.5, +1) | -29.1(-32.6, -25.6) |
|  | Overweight | -27.7(-29.0, -26.5) | -3.4( -5.2, -1.5) | -31.1(-32.8, -29.4) |
|  | Obese I | -28.8(-29.9, -27.6) | -3.2(-4.9, -1.3) | -32.9(-34.7, -31.0) |
|  | Obese II | -29.2(-30.7, -27.7) | -2.3(-4.5, -0.0) | -31.5(-33.6, -29.3) |
|  | Obese III | -29.3(-30.8, -27.7) | -1.7(-4.2, +0.8) | -31.0(-33.3, -28.6) |
| Mean improvement= -31.1 | | | | |
| **Pain †** | | | | |
|  | Normal | -24.8(-27.3), -22.2, | -5.2(-9.0, -1.2) | -29.9(-33.5, -26.3) |
|  | Overweight | -26.7(-28.0, -25.4) | -6.0(-7.9, -4.0) | -32.7(-34.4, -30.8) |
|  | Obese I | -27.8(-29.0, -26.5) | -5.1(-7.0, -3.2) | -34.3(-36.2, -32.4) |
|  | Obese II | -29.1(-30.7, -27.5) | -2.9(-5.4, -0.4) | -32.0(-34.2, -29.7) |
|  | Obese III | -29.6(-31.3, -27.9) | -3.3(-6.0, -0.6) | -32.9(-35.4, -30.4) |
| Mean improvement = -32.4 | | | | |
| **Function†** | | | | |
|  | Normal | -27.3(-29.7, -24.9) | -1.8(-5.5, -1.9) | -29.1(-32.6, -25.6) |
|  | Overweight | -29.1(-30.3, -27.9) | -2.0(-3.9, -0.2) | -31.1(-32.8, -29.4) |
|  | Obese I | -30.1(-31.2, -28.9) | -1.9(-3.7, -0.1) | -32.9(-34.7, -31.1) |
|  | Obese II | -30.4(-31.9, -28.9) | -1.0(-3.3, 1.3) | -31.5(-33.6, -29.3) |
|  | Obese III | -30.5(-32.1, -28.9) | -0.6(-3.1, +2.0) | -31.1(-33.5, -28.7) |
| Mean improvement = -31.1 | | | | |
| **Stiffness†** | | | | |
|  | Normal | -20.2 (-23.0, -17.3) | -7.3(-11.7, -2.9) | -27.5(-31.6, -23.4) |
|  | Overweight | -22.0(-23.4, -20.5) | -6.6(-8.8, -4.5) | -28.6(-30.6, -26.6) |
|  | Obese I | -22.5(-23.9, -21.1) | -7.3(-9.4, -5.2) | -30.1(-32.1, 28.0) |
|  | Obese II | -23.9(-25.6, -22.1) | -4.2(-6.9, -1.5) | -28.1(-30.6, -25.6) |
|  | Obese III | -23.1(-25.0, -21.3) | -5.1(-8.1, -2.2) | -28.3(-31.0, -25.5) |
| Mean improvement = -28.5 | | | | |
| **EQ5D**Ψ | | | | |
|  | Normal | 0.24 (0.20, 0.28) | 0.01(-0.06, 0.08) | 0.23(0.17, 0.30) |
|  | Overweight | 0.26(0.24, 0.28) | 0.01(-0.04, 0.03) | 0.26(0.23, 0.30) |
|  | Obese I | 0.26(0.24, 0.28) | 0.01(-0.04, 0.03) | 0.27(0.23, 0.30) |
|  | Obese II | 0.29(0.27, 0.32) | 0.01(-0.04, 0.05) | 0.29(0.24, 0.33) |
|  | Obese III | 0.30(0.28, 0.33) | 0.01(-0.05, 0.04) | 0.31(0.27, 0.35) |
| Mean improvement = 0.27 | | | | |
| Negative mean changes for WOMAC scores (total, pain, function, and stiffness) indicate improvement. **†** Scale of 0 to 100, with 100 being the worst. Ψ1.00 indicating full health and 0 representing death. | | | | |
